# Speckle tracking echocardiography: another step towards early detection of septic myocardial dysfunction?

**DOI:** 10.1186/s13054-016-1411-5

**Published:** 2016-08-18

**Authors:** Elio Antonucci, Sara Agosta

**Affiliations:** Intermediate Care Unit, Emergency Department, Guglielmo da Saliceto Hospital, Piacenza, Italy

We read with interest the recent paper by Ng et al. [1] dealing with the use of speckle tracking echocardiography (STE) in septic myocardial dysfunction. In this study, the authors found that STE can detect a left ventricular impairment more sensitively than conventional echocardiography in septic shock patients. The study offers new perspectives to improve diagnosis of septic myocardial dysfunction, and the authors should be commended for their work. However, some important hemodynamic issues should be further discussed to better interpret the results.

Firstly, the cardiac preload was represented by the right atrial pressure (RAP), estimated by the inferior vena cava (IVC) diameter and the presence of inspiratory collapse. Results proved that the RAP was higher in the study group of patients than in the control group (7.4 mmHg vs 5.9 mmHg, *p* = 0.017), even if the amount of fluids given in the first hours of shock were not known. Moreover, if we consider that only 16/33 patients (48 %) were mechanically ventilated, use of the IVC diameter could be debatable to predict the cardiac preload in spontaneous breathing patients, as reported previously [2].

Secondly, no information about the dosage and type of vasopressor/inotropic support was provided. In patients supported by catecholamines, STE might not be accurate because of altered strain rates induced by high doses of dobutamine [3].

In addition, mean cardiac output (CO) and the afterload measures were unexpectedly similar both in the study group and in the control group of patients (CO = 5.88 L/min vs 5.48 L/min, *p* = 0.40; SVR = 1090 dynes•sec/cm^5^ vs 1194 dynes•sec/cm^5^, *p* = 0.303) despite the presence of distributive shock in the first group. Moreover, relevant data about tissue perfusion and microcirculatory dysfunction, such as lactate levels and venous-to-arterial carbon dioxide differences, were not reported.

Lastly, the authors evaluated both mechanically ventilated patients and nonventilated patients in the study group. We know that the effects of mechanical ventilation on cardiac function and hemodynamics are complex and are characterized by reduced venous return (due to an increase in RAP), increased pulmonary vascular resistance (PVR), and reduced CO [4]. However, the present study is lacking in some important information about the positive end-expiratory pressure (PEEP), tidal volume, and respiratory rate, data that, instead, were accurately reported in the previous echocardiography works in this field [5].

In conclusion, further prospective studies are warranted to investigate the clinical utility of STE in septic myocardial dysfunction.
