# Supplementary material for: Speckle tracking echocardiography: another step towards early detection of septic myocardial dysfunction?
Source: Crit Care. 2016 Aug 18;20:236. doi: 10.1186/s13054-016-1411-5 (PMC4989354; doi:10.1186/s13054-016-1411-5)
Supplement: Related texts

# Reply: Authors’ response

**Authors:** Pauline Yeung Ng, Wai Ching Sin, Andrew Kei-yan Ng

We thank Dr Antonucci and Dr Agosta for their interest in our research article [1] and their comments.

We reported RAP estimated by the IVC diameter and collapsibility as a measure of cardiac preload. Although a difference in RAP between the two groups existed (7.4 mmHg vs 5.9 mmHg, *p* = 0.017), the absolute difference of 1.5 mmHg is probably clinically negligible. Left ventricular end diastolic volume (LVEDV) is a better index of LV preload [6], and was similar in the two groups (74.1 ml vs 74.7 ml, *p* = 0.90), suggesting similar preload conditions. The effect of preload on strain parameters is not completely understood, but small-scale studies have shown cardiac strain to be relatively load independent [7].

Regarding the use of vasopressor or inotropic support, all patients received noradrenaline as the initial drug of choice during resuscitation. No patients received dobutamine during the course of treatment.

We attribute the similar CO and systemic vascular resistance (SVR) in the study and control groups to two factors. Firstly, instead of healthy individuals, the control group was a group of patients with sepsis. These patients would have similarly raised CO and reduced SVR, albeit to different degrees. Secondly, because of the time-consuming nature of performing strain analysis, all echocardiographic assessments were done after initial stabilization, which included vasopressor administration in the context of the distributive shock. Although logistically difficult, it will be an excellent research topic to assess the speckle tracing during the initial period of septic shock.

Finally, the effect of mechanical ventilation on hemodynamics was not explored in depth in our study. Subgroup analysis was not performed due to the relatively small number of patients (16/33) on mechanical ventilation. However, a similar right ventricular index of myocardial performance between the two groups speaks against a significant effect of mechanical ventilation on right ventricular function.

## Abbreviations

CO, cardiac output; IVC, inferior vena cava; LVEDV, left ventricular end diastolic volume; PEEP, positive end-expiratory pressure; PVR, pulmonary vascular resistance; RAP, right atrial pressure; STE, speckle tracking echocardiography; SVR, systemic vascular resistance
